# College Medical Dispensary

**Published:** 1847-07

**Authors:** 


ART. III. *College Medical Dispensary.—Case Morbus Cordis.*

The following case, wlth clinical remarks, is selected from those which have occurred at the College Medical Dispensary since the former report, because it illustrates points connected with valvular disease of heart, to which reference is made in the foregoing review of Latham's work.

——— *Castle*, aged about 40, soldier, has lately returned, on sick leave, from Mexico. He has Pharyngitis, associated with chronic Laryngitis, producing hoarseness, some cough, and paroxysms of spasmodic contraction of the muscles of the pharynx and glottis, excited by the deglutition of liquids, so that they are at times expelled with force through the nostrils. I cannot satisfy myself of the existence of pulmonary disease. He has, also, a large nodosity in the neighborhood of the right knee-joint.

But the feature of greatest interest in this case is the presence of a loud bellows' murmur in the heart. This murmur is of a soft quality, and occurs at the termination of the first, or systolic sound. At what orifice is it produced? We are to determine this point by the timbre, or pitch of the murmur, the point in the præcordial region where exists its maximum of intensity, and the directions from that point in which the murmur continues farthest, and most audibly. Now the murmur is manifestly loudest over the apex of the heart —here is its maximum—it continues to be heard distinctly for some distance *below* this point, It is heard also over the body of the organ, but between the second and third ribs, over the sigmoid valves, it is indistinct, and disappears upon carrying the stethoscope *above* this point. Its pitch is low, approaching nearer to *who* whispered, than to *R*. From these circumstances it is to be inferred that the morbid sound is produced at the *mitral valve*, not at the aortic orifice.

Does it proceed from regurgitation through the mitral valve? The answer to this question is affirmative, because it occurs in connection with the first, or systolic sound. If the murmur were occasioned by the passage of the blood in its natural course through the left auriculo-ventricular orifice, it would occur with the diastolic or second sound. Besides, an audible murmur from this cause is extremely rare, while it is caused by regurgitation in the larger proportion of the cases in which this sign is present.

It is referred to the mitral, rather than to the tricuspid valve by the law of probalitities, the former being affected in the vast majority of cases.

We are then justified in diagnosticating a lesion of the mitral valve sufficient to permit regurgitation through the orifice which this valve, in its healthy condition, protects.

Let us now direct attention to the clinical history, and pathological relations of the heart affection in this case. The patient from early life has been subject to acute rheumatism. He is not aware that inflammation of the heart has ever complicated any of his rheumatic attacks. Does not remember that he ever suffered pain in the chest at those times, nor was medical treatment ever directed to the region of the heart. He has, however, doubtless, at some former period, suffered from endocarditis, which has left the endocardium in a damaged condition. How long it may be, since its occurrence it is impossible to say—perhaps many years have elapsed—the affection is probably of an old date. Whenever it occurred, it was at the time overlooked, from the absence of any obvious symptoms denoting the heart to be the seat of an inflammatory disease. The inflammation was of a low grade—subacute—and had it been recognized, the permanent injury perhaps could not have been averted, as it follows in a large proportion of cases, whatever measures are taken to prevent it.

The patient up to this time, and now, makes no complaint of any heart affection. He is conscious of no trouble, nor of any uncomfortable sensations in the præcordial region; has no palpitation, dyspnœa, nor other palpable evidences of impeded circulation in the organ. The impulse is not much, if at all increased, and the rhythm is natural. I observed on making an examination in the recumbent position, that on first lying down, there was considerable irregularity, which passed away in a few moments. His own sensations did not render him conscious of the irregularity.

The regurgitation has not, as yet, led to much hypertrophy, which is almost necessarily a result sooner or later. Probably the retrograde current is small in amount, and does not occasion much stagnation in the left auricle, and pulmonary vascular system. If it were otherwise, we should have evidences of the fact, not only in palpable signs connected with the heart itself, but in consecutive disorders of the pulmonary system, &c.,

This case illustrates that the bellows' murmur, even denoting regurgitation, is not necessarily a sign of very serious disease. It has existed in this patient probably for years, and it may be many years before impediment to the circulation is produced sufficiently to occasion distressing symptoms, or fatal accidents. If not cut off by other disease he may expect ultimately to suffer from well declared heart disease, which, either by itself, or by means of secondary affections, will shorten life.

The present condition of the heart calls for no medical treatment. There is no progressive disease there now, but only the irremediable results of former disease. It is, however, highly important for the patient's welfare that he avoid all causes which tend to excite the heart's action, to surcharge the vascular system, or to obstruct the circulation in any part of the system.

The Pharyngitis and Laryngitis are the affections now claiming medical attention. These I propose to treat by the topical application of a strong solution of the nitrate of silver, (ʒi to ℥i).

---

*Case of Aneurism of the Aorta descendens producing symptoms of gastralgia.*

Our readers will have observed among the cases reported in the May No. of vol. 2d, as occuring at the college Medical Dispensary, a case entitled *gartralgia*, a brief account of the history of which was given—see the No. referred to.

This patient subsequently came under the care of Dr. Cary, of this city, to whom is due the credit of detecting the source of the ailments which have incapacitated him for labor and comfort for more than a year past. On careful exploration of the abdomen, Dr. C., ascertained the existence of an aneurism, probably of the descending aorta. A distinct, although not very strong pulsation is perceptible just above the umbilicus. Whether this was noticed at my examination in November last I cannot say, not having kept any memorandum of that examination. The patient states that it existed a year ago. At all events, I failed to recognize the tumor, and I confess did not entertain suspicion of an aneurism. Upon examination, by request of Dr. Cary, I am satisfied that his diagnosis is correct. The tumor can be isolated both by the pulsation and by manual compression. It is globular, smooth like a cyst, and moveable from side to side. There is, also, a distinct *bruit de souffle*, or bellows' murmur, heard loudest just above the tumor. Here, then, we have the *sedes malorum* of the ailments which, in our general examination, appeared indefinite and obscure. Unfortunately the light shed on the case by the closer examination, and the tact of Dr. C., does not afford a more encouraging view of its remediable character. As often happens, a correct diagnosis takes away expectations of cure which previously may have been entertained. I must cheerfully and gladly, however, embrace an opportunity to refer to the case, as well to do justice to Dr. Cary, as to enforce the great importance of exploring carefully, in all cases of like obscurity, for some palpable, localized lesions. There is a scientific satisfaction in thus tracing effects to causes, albeit we shall be unable to add to it the more agreeable office of encouraging our patients with the prospect of recovery.

